# Targeting HCCR expression resensitizes gastric cancer cells to chemotherapy via down-regulating the activation of STAT3

**DOI:** 10.1038/srep24196

**Published:** 2016-04-07

**Authors:** Jun-Ling Zhang, Xiang-Zheng Liu, Peng-Yuan Wang, Guo-Wei Chen, Yong Jiang, Shu-Kai Qiao, Jing Zhu, Xin Wang, Yi-Sheng Pan, Yu-Cun Liu

**Affiliations:** 1 Department of Thoracic Surgery, Peking University First Hospital, Beijing 100034, P. R. China; 2 Department of General Surgery, Peking University First Hospital, Beijing 100034, P. R. China; 3 Department of Hematology, The Second Hospital of Hebei Medical University, Shijiazhuang, 050000, P. R. China

## Abstract

The human cervical cancer oncogene (HCCR) has been found to be overexpressed in a variety of human cancers. However, the level of expression of HCCR and its biological function in gastric cancer are largely unknown. In this study, we evaluated HCCR expression in several gastric cancer cell lines and in one normal gastric mucosal cell line. We established a 5-FU-resistant gastric cancer cell subline, and we evaluated its HCCR expression. HCCR expression levels were high in gastric cancer lines, and expression was significantly increased in the 5-FU-resistant cancer cell subline. HCCR expression affected cell growth by regulating apoptosis in the cancer cells, and it had a positive correlation with p-STAT3 expression. Western blot and luciferase reporter assays showed that the activation of STAT3 upregulated HCCR expression in a positive feedback loop model. *In vivo* and *in vitro* studies showed that HCCR plays an important role in the apoptosis induced by 5-FU. Our data demonstrate that HCCR is probably involved in apoptosis and cancer growth and that it functions as a p-STAT3 stimulator in a positive feedback loop model. In gastric cancer cells, HCCR confers a more aggressive phenotype and resistance to 5-FU-based chemotherapy.

Gastric cancer is the second most common cancer in China and represents the second leading cause of cancer deaths (738,000 deaths, 9.7%) worldwide^1^,^2^. Almost two-thirds of gastric cancer cases occur in developing countries^1^,^2^. With the introduction of adjuvant therapy followed by surgeries, considerable improvements have been made in the outcomes of patients with gastric cancer. However, the prognosis for patients with gastric cancer is still poor because of chemotherapy resistance^3^. Thus, how to resensitize gastric cancers to chemotherapy remains a significant clinical challenge.

The human cervical cancer oncogene (HCCR), also known as LETM1 domain containing 1 (LETMD1), was isolated by Ko in cervical cancer cell lines by using differential display reverse transcriptase-PCR (DDRT-PCR)^4^. The HCCR gene is located in the long arm of chromosome 12 and is classified according to its molecular characteristics as one of two isoforms, HCCR1 or HCCR2. A comparison of both sequences has revealed that HCCR2 lacks exon 1 of HCCR1^4^. HCCR1 encodes a molecule with 360 amino acids (~42 kDa) and HCCR2 encodes a molecule with 304 amino acids (~36 kDa)^4^. In addition, Northern and western blot analyses revealed that fresh primary human gastric cancer tissues showed increased expression of HCCR compared with their normal counterparts^5^. HCCR was found to be overexpressed in various human malignancies, such as leukaemia/lymphoma and breast, kidney, colon, liver and ovarian cancer^4^. In addition, it has been found that HCCR might be a novel oncogene that is involved in tumourigenesis and may function as a negative regulator of the p53 tumour suppressor^5^. Transgenic mice expressing HCCR developed metastatic breast cancer, and the levels of p21^WAF1^, MDM2, and bax decreased in the spontaneous breast cancer cells and metastatic tumours of HCCR transgenic mice^6^. Serological studies revealed an 86.8% sensitivity for HCCR in patients with breast cancer, which was higher than the 21.0% for CA15-3^7^. Our previous study found that HCCR plays an important role in regulating apoptosis and the cell cycle in leukaemia^8^.

STAT3 is known to promote cell proliferation and angiogenesis and play a role in the invasiveness and metastatic potential of cancers. STAT3 activation has been reported in nearly 70% of solid and haematological tumours, including gastric cancer, breast cancer, colorectal cancer, lymphomas, multiple myeloma, and leukaemia. Phospho-STAT3 overexpression is a prognostic factor of patients with digestive system cancers^9^. The activation of STAT3 promotes gastric cancer progression, suppresses apoptosis and promotes invasion^10^.

At present, the primary function of HCCR in gastric cancers remains unclear. We hypothesized that HCCR might modulate cell proliferation and apoptosis by regulating the activation of STAT3 in gastric cancer cells. Therefore, the aim of the present study was to explore HCCR expression and characterize its function in gastric cancer cell lines. Moreover, we explored its possible molecular mechanism in relation to chemoresistance.

## Results

### Gastric cancer cell lines expressed high levels of HCCR

**Figure 1:**
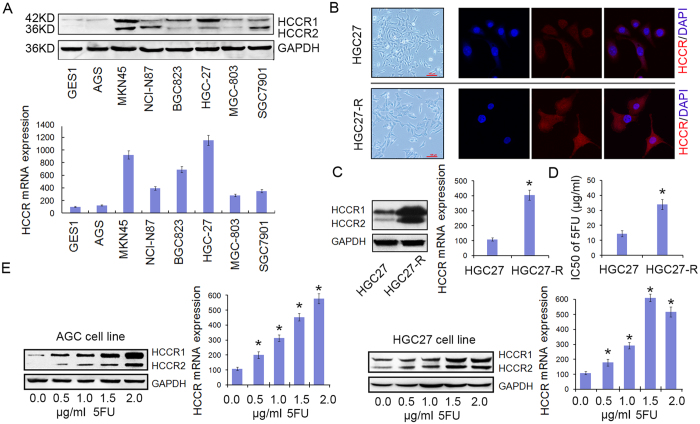
Gastric cancer cell lines expressed high levels of HCCR, which was associated with 5-FU resistance. (**A**) HCCR protein expression (upper panel) and mRNA expression (lower panel) in 7 types of gastric cancer cell lines and 1 normal gastric cell line. (**B**) Morphologies of HGC-27 (upper panel) and HGC-27-R (lower panel) cells (40×). HCCR immunofluorescence staining is shown in red, and the nuclei are counter-stained with DAPI (blue). (**C**) Western blot and qPCR detection of HCCR expression in HGC-27 and HGC-27-R cells. *P = 0.0085. (**D**) The IC50 values of 5-FU for HGC-27 and HGC-27-R cells. *P < 0.001. (**E**) AGS and HGC-27 cells were cultured in the presence of various concentrations of 5-FU (0.0, 0.5, 1.0, 1.5 or 2.0 μg/mL) for 6 h. The western blot and qPCR assays detected HCCR expression. All results are shown as the means (±s.d.) of three separate experiments, *P < 0.01.

The western bolt and qPCR assays showed that SGC-7901, MGC-803, NCI-N87, and BGC-823 expressed median levels of HCCR and that HGC-27 and MKN45 expressed high levels of HCCR. Compared with the cancer cell lines, the normal gastric mucosa epithelial cell line GES-1 expressed almost no HCCR (Fig. 1A).

### HCCR expression was significantly upregulated in the 5-FU-resistant gastric cancer cell subline

The 5-FU-resistant gastric cell subline HGC-27-R was developed from the parental HGC-27 cell line. As observed by optical microscopy, HGC-27-R cells had a large swollen form compared with the parental HGC-27 cells. To test whether HCCR plays a role in the acquired 5-FU resistance of HGC-27-R cells, HCCR expression in HGC-27-R cells was detected. The immunofluorescence staining showed that HGC-27-R cells expressed high levels of HCCR (Fig. 1B). In addition, the western blot and qPCR assays showed that the HCCR expression in HGC-27-R cells was significantly increased by approximately 4-fold compared with that in parental HGC-27 cells (P = 0.0085; Fig. 1C). We showed that the IC50 of 5-FU for HGC-27 and HGC-27-R cells was (13.31 ± 2.57) μg/mL and (31.76 ± 3.16) μg/mL, respectively (P < 0.001; Fig. 1D), suggesting that the HGC-27-R cell line was approximately 2.39 times more resistant than the parental cell line. To further verify the critical role of HCCR in the process of acquiring 5-FU resistance, western blot and qPCR assays were performed to detect HCCR expression in AGS and HGC-27 parental cells treated with various concentrations of 5-FU (0.0, 0.5, 1.0, 1.5 and 2.0 μg/mL) for 6 hours. The results showed that the relative levels of HCCR expression were significantly increased (P < 0.01; Fig. 1E). However, the apoptotic rates of these cells were not changed significantly (data not shown). Thus, an increasing level of HCCR expression in gastric cancer cells was induced by a low concentration of 5-FU over a brief treatment period. In conclusion, HCCR up-regulation may be an important part of acquiring 5-FU resistance.

### HCCR expression rescued cells from 5-FU induced apoptosis

**Figure 2:**
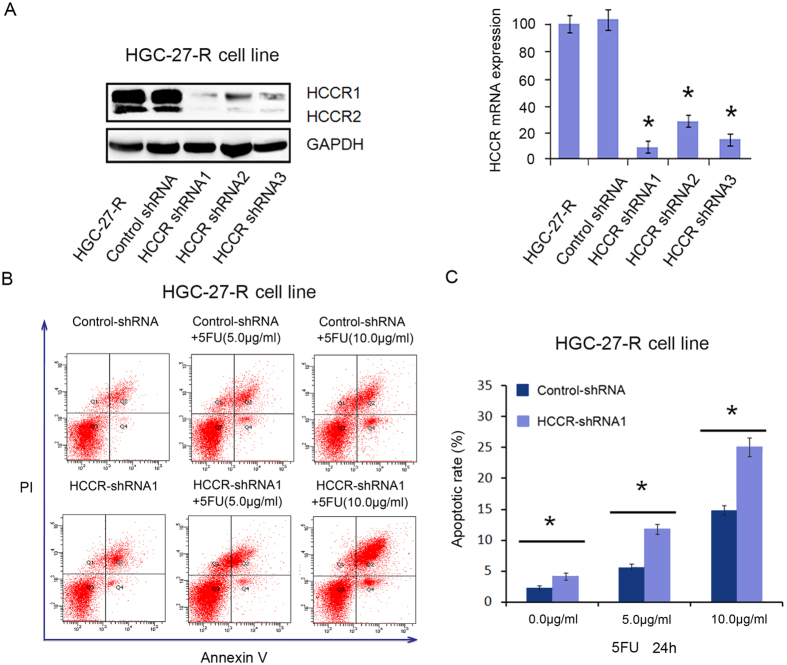
HCCR silencing significantly decreased the resistance of HGC-27-R cells to 5-FU. (**A**) The western blot and qPCR detection of HCCR expression in HGC-27-R cells stably transfected with a negative control or 3 different HCCR-shRNAs. *P < 0.001. (**B,C**) FCM analysis of the 5-FU-induced rate of apoptosis in HGC-27-R cells transfected with control shRNA or HCCR-shRNA1. Cells in the media were treated with progressive concentrations of 5-FU (0.0, 5.0 or 10.0 μg/mL) for 24 h before the FCM assay. All results are shown as the means (±s.d.) of three separate experiments, *P < 0.01.

To further investigate the effect of HCCR expression on the sensitivity of gastric cancer cells to 5-FU, resistant HGC27-R cells were transfected with control shRNA, HCCR-shRNA1, HCCR-shRNA2 or HCCR-shRNA3. After 48 h of transfection, western blot and qPCR assays were performed to detect the expression of HCCR. The data revealed that all 3 HCCR-shRNAs significantly inhibited HCCR expression levels (P < 0.001; Fig. 2A). As HCCR-shRNA1 showed the highest efficiency in silencing HCCR expression, we selected HCCR-shRNA1 for the subsequent experiments. We further investigated HCCR role and mechanism of action in 5-FU resistance. When HGC27-R cells were transfected with HCCR-shRNA1 combined with 5-FU treatment (0.0, 5.0 and 10.0 μg/mL) for 24 hours, it was found that the HCCR-shRNA1-mediated downregulation of HCCR significantly increased the 5-FU-induced apoptosis of chemotherapy-resistant cancer cells (P < 0.01; Fig. 2B, C).

### HCCR knockdown inhibited cell growth rates and induced apoptosis

**Figure 3:**
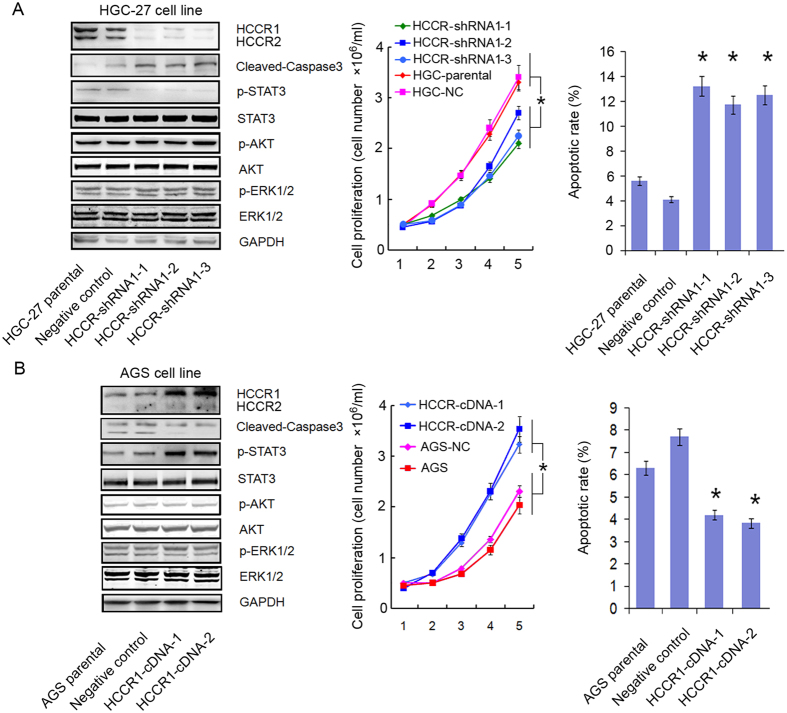
HCCR silencing inhibited cell growth and induced apoptosis by modulating the expression of p-STAT3. Protein expression was determined by western blot assay, cell proliferation was assayed by the MTT method, and apoptosis was assayed by FCM. (**A**) Parental gastric cancer cells (red) and negative control cells (pink) showed more growth than HCCR-shRNA1-transduced cell clones (navy, light blue or green). *P < 0.001. Apoptosis in parental cells was significantly lower than in HCCR-shRNA1-transduced cells. *P < 0.001. (**B**) The proliferation of HCCR1-cDNA-transduced AGS cells (navy or light blue) was significantly higher than that of parental cells (red) and negative control cells (pink) *P < 0.001. Apoptosis in HCCR1-cDNA-transduced cells was significantly lower than in parental cells, *P < 0.001.

To further test the roles of HCCR overexpression in the development of 5-FU resistance in cancer cells, HCCR-shRNA-1 was stably transfected into HGC-27 cells. We developed and selected three subclones, which were named shRNA1-1, shRNA1-2, and shRNA1-3. Compared with the expression in the negative control cells, the level of HCCR expression in HCCR-shRNA1 transfected clones was decreased (Fig. 3A). Additionally, we observed that the down-regulation of HCCR could significantly decreased the growth rate of cancer cells by approximately 73.2% (P < 0.001; Fig. 3A). Additionally, the FCM assay showed that the down-regulation of HCCR could lead to increased rates of apoptosis in cancer cells (P < 0.001; Fig. 3A). To confirm the above results, the cDNA of HCCR1 was stably transfected into the AGS cell line, and the level of HCCR1 expression was increased significantly (P < 0.001; Fig. 3B). The upregulation of HCCR1 also significantly increased the growth rate of AGS cells by approximately 3.42-fold. Likewise, the upregulation of HCCR1 induced a decreased rate of apoptosis in AGS cells (P < 0.001; Fig. 3B).

### HCCR regulated the activation of STAT3 and caspase-3

It is believed that p-STAT3 modulates apoptosis in cancer cells and cleaved caspase-3 levels, which is a marker of apoptosis. Thus, a western blot assay was performed to detect the effect of HCCR expression on the activity of STAT3 and caspase-3. As shown in Fig. 3, the downregulation of HCCR led to a decrease in p-STAT3 protein expression in HGC-27 cells, whereas the expression of cleaved caspase-3 was significantly increased. As expected, HCCR1 upregulation led to the activation of STAT3 in AGS cells. Correspondingly, cleaved caspase-3 expression was decreased significantly. However, we did not find that HCCR expression affected the activity of ERK1/2 or AKT. Further study showed that neither HCCR silencing nor forced expression affected the expression of P21 and P27 (data not shown).

### The apoptosis of cancer cells modulated by HCCR expression was dependent on STAT3 activity

**Figure 4:**
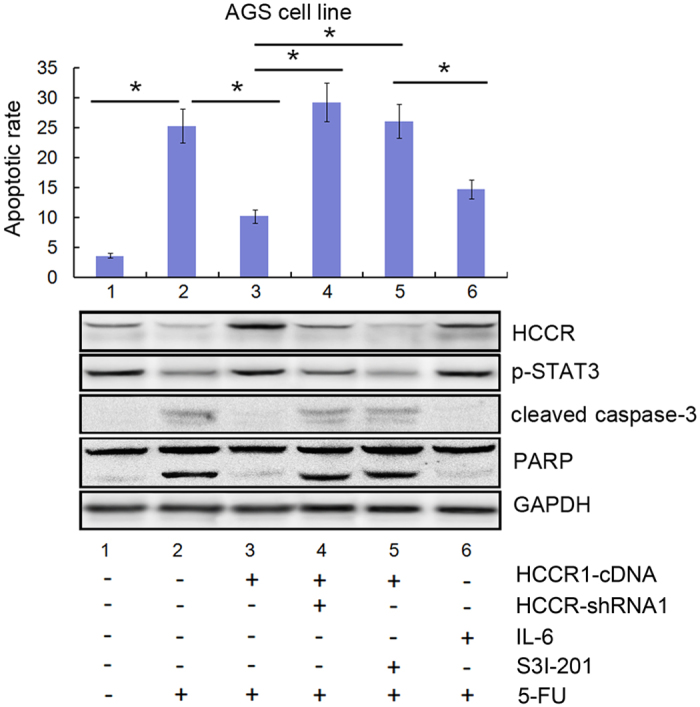
The 5-FU-induced apoptosis modulated by HCCR expression was dependent on STAT3 activity. HCCR-shRNA/HCCR1-cDNA-transduced AGS cells were treated with IL-6, a STAT3 inhibitor (S3I-201), and 5-FU (10.0 μg/mL) for 24 h. Cell apoptosis was determined by FCM assay. HCCR, p-STAT3, cleaved caspase-3 and PARP levels were assayed by western blot. The results are shown as the means (±s.d.) of three separate experiments, *P < 0.05.

To further study the potential mechanism by which HCCR regulates apoptosis, we explored the role of STAT3 in the apoptosis induced by 5-FU using AGS cell line. As shown in the Fig. 4, we used a pcDNA3.1 vector carrying HCCR1 cDNA, HCCR-shRNA, IL-6 or a STAT3 inhibitor (S3I-201) to effect HCCR and p-STAT3 expression. The results of the FCM analysis showed that HCCR1 overexpression decreased the rate of 5-FU-induced apoptosis and that p-STAT3 expression was increased while PARP and cleaved caspase-3 expression was decreased (line 3). When HCCR was knocked down by shRNA, these phenomena disappeared (line 4). As shown in line 5, when p-STAT3 expression was blocked by S3I-201 in HCCR1-overexpressing AGS cells, the apoptotic rate was increased significantly. Moreover, compared with the HCCR levels of cells transfected by HCCR1-cDNA, the expression of HCCR was repressed by the STAT3 inhibitor. As shown in line 6, when IL-6 was used to increase the expression of p-STAT3, the expression of HCCR was correspondingly increased. At the same time, the apoptotic rate of the cells was decreased by IL-6 treatment. As expected, the expression of cleaved caspase-3 and PARP was decreased. In summary, the apoptosis of cancer cells regulated by HCCR expression was dependent on STAT3 activity (Fig. 4).

### The activation of STAT3 upregulated the expression of HCCR in a positive feedback loop model

**Figure 5:**
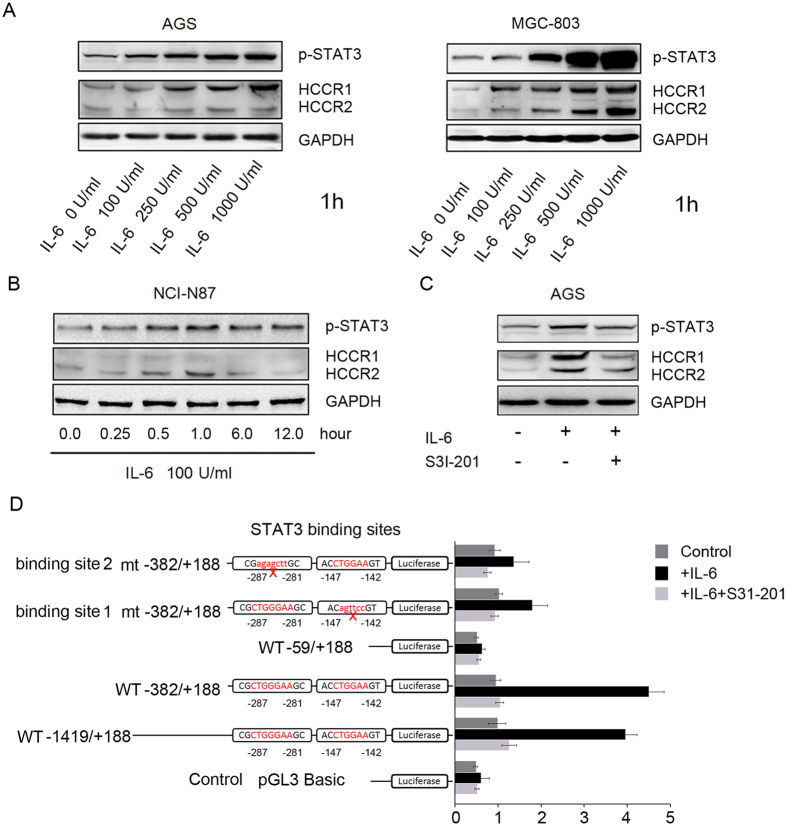
The activation of STAT3 regulated the expression of HCCR. (**A**) AGS and MGC-803 cells were treated with various concentrations of IL-6 for 1 h. HCCR expression increased as the IL-6 concentration increased. (**B**) The NCI-N87 cells were treated with 100 IU/mL IL-6 for 0.25 h, 0.5 h, 1.0 h, 6.0 h, and 12 h. An increase in HCCR expression was rapidly detectable within 0.5 h. The increase in p-STAT3 was detectable within 0.25 h and was lasted for 12 h. (**C**) IL-6 increased the expression of HCCR, but HCCR expression was blocked by the STAT3 inhibitor S3I-201. (**D**) Schematic drawing representing the step-by-step deletion of the HCCR-promoter luciferase (Luc)-reporter constructs as well as mutant (mt)- and wild type (WT)-reporter constructs. Relative locations of the putative binding sites are shown. Cells were transfected with different reporters for 24 h and were treated with or without IL-6 and its inhibitor (S31-201). Luciferase reporter activities were detected. The results are shown as the means (±s.d.) of three separate experiments.

In the previous experiments, we demonstrated that HCCR expression could be decreased by a STAT3 inhibitor (S3I-201). Correspondingly, HCCR expression increased when IL-6 was used to stimulate the activity of STAT3. To confirm these results, we treated AGS and MGC-803 cells for one hour with various concentrations of IL-6. Marked p-STAT3 and HCCR protein induction as a result of IL-6 treatment was detectable at doses as low as 100 IU/mL (Fig. 5A). Then, we treated NCI-N87 cells for various times using a medium with 100 IU/mL IL-6. The effect was detectable within 30 min, demonstrating that IL-6 is a potent inducer of the HCCR protein. Importantly, the induction of p-STAT3, which was detectable within 15 min and prolonged for at least 12 hours, preceded the induction of HCCR (Fig. 5B). Others have previously demonstrated that IL-6 is capable of activating p-STAT3, but it was unknown whether p-STAT3 could modulate the expression of HCCR. Our study shows that IL-6 could increase the expression of HCCR and p-STAT3 in AGS cells. However, when the STAT3 inhibitor S3I-201 (50 μM) was used, the expression of HCCR was repressed (Fig. 5C). These phenomena demonstrate that the activation of STAT3 upregulates the expression of HCCR in a positive feedback loop model.

### STAT3 activity was required for the activation of the HCCR promoter

To define the molecular motifs within the HCCR promoter responsible for STAT3 activation, several luciferase reporter constructs containing various lengths of the 5′ upstream proximal promoter region of HCCR were designed (Fig. 5D), and luciferase activity was assessed. As shown in Fig. 5D, the full-length reporter (−1419/+188-Luc) increased luciferase activity ~4-fold after treatment with IL-6 (100 IU/mL) for 1 hour, while the activity of the −382/+188-Luc reporter construct was stimulated by ~4.5-fold compared to the activity of the control plasmids. Meanwhile, the −59/+188-Luc reporter construct, which lacked the distal 323-bp region of the −382/+188-Luc, lost its ability to respond when stimulated by IL-6. Computational analysis indicated the presence of two putative STAT3 binding sites within this region (−142 to −147 bp and −281 to −287 bp), which led us to further investigate whether STAT3 was involved in HCCR transcription. Elimination of the binding site by point mutation was performed in the −382/+188-Luc construct. As shown in Fig. 5D, the core sequences in the two binding sites were changed, and luciferase activities of the two STAT3-mutated reporter constructs were significantly decreased (Fig. 5D). Furthermore, all assays showed that luciferase activity could be inhibited by the STAT3 inhibitor S3I-201.

### HCCR promoted gastric cancer growth *in vivo*

**Figure 6:**
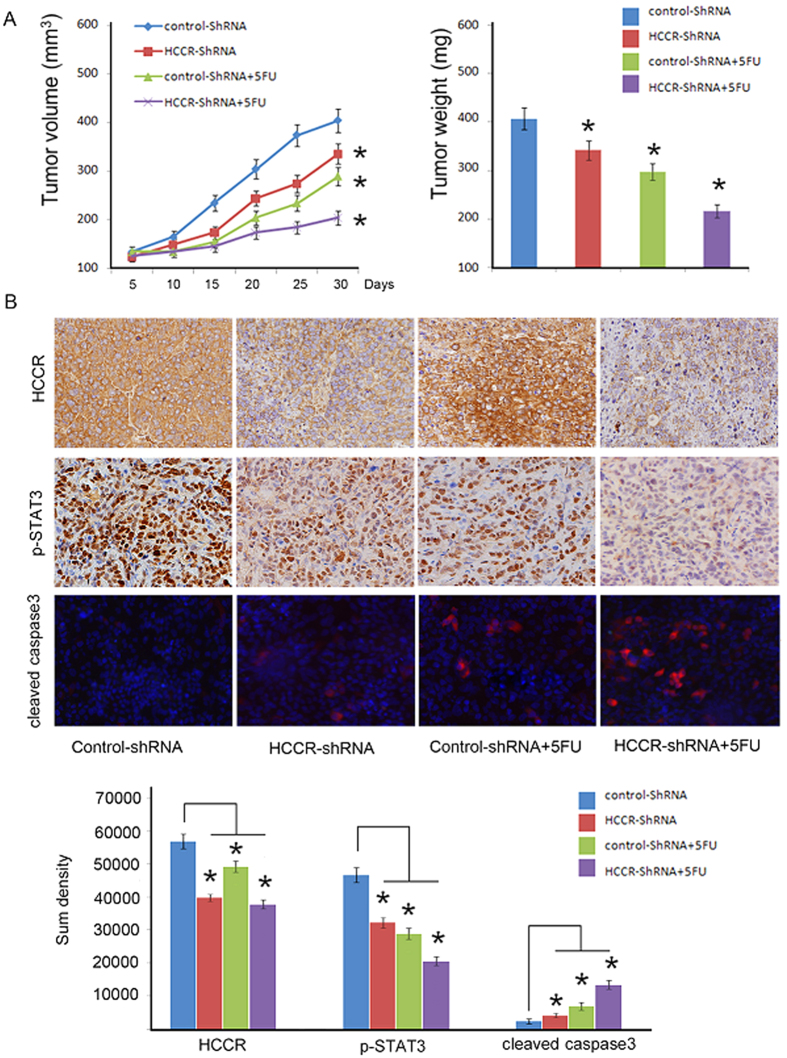
HCCR silencing repressed gastric cancer progression *in vivo* and sensitized gastric cancer cells to 5-FU. HCCR-shRNA and negative control cells were subcutaneously injected into nude mice that were/were not treated with 5-FU. (**A**) The volume and weight of the tumours are indicated by the mean values (±s.d.) of six mice from each group, *P < 0.05, significant difference from control cells. (**B**) The sections were stained with antibodies against human HCCR, p-STAT3 and cleaved caspase-3. The sum densities were calculated and analysed with IPP 6.0. Columns are means (±s.d.) of quintuplicate determinations of six mice from each group. *P < 0.05.

HCCR-shRNA1 and negative control shRNA cells were subcutaneously injected into the mice that were/were not treated with 5-FU. The tumour growth curves and harvested tumour weights are shown in Fig. 6A. Tumour growth was significantly decreased in the HCCR-shRNA1 group compared to growth in the control group (P = 0.032; Fig. 6A). An even more significant reduction was observed in the HCCR-shRNA1 group treated with 5-FU (P = 0.002; Fig. 6A). The tumour weights were also significantly decreased in the HCCR-shRNA1 group (P = 0.043; Fig. 6A). An even more significant reduction was observed in the HCCR-shRNA1 group treated with 5-FU (P = 0.004; Fig. 6A). HCCR and p-STAT3 expression levels in tumours developed from HCCR-shRNA1-transfected cells were significantly lower than in those from control-shRNA-transfected cells (P < 0.05; Fig. 6B). However, cleaved caspase-3 expression was increased (P < 0.05; Fig. 6B). Following treatment with 5-FU, the HCCR and p-STAT3 levels in tumours developed from HCCR-shRNA1-transfected cells were significantly decreased compared with those in the tumours developed from control shRNA-transfected cells (P < 0.05; Fig. 6B). Cleaved-caspase-3 staining indicated that the rate of apoptosis in cells was significantly increased in the tumours developed from HCCR-shRNA-transfected cells (P < 0.05; Fig. 6B).

## Discussion

Tumour chemoresistance remains one of the most significant challenges to the successful treatment of gastric cancer. Currently, the majority of cancer patients who show an initial response to treatment will eventually develop aggressive malignancies. Such malignant cells may exhibit up to 90% resistance to one or more drugs. Despite the clinical prevalence of resistance to chemotherapeutic agents, the underlying mechanisms of resistance are still poorly understood.

HCCR proteins have been implicated in a variety of functions, including oncogenesis. A previous study found that HCCR was overexpressed in the serum of patients with hepatocellular carcinoma and indicated that the HCCR assay has an advantage over the alpha-fetoprotein (AFP) assay in the early diagnosis of hepatocellular carcinoma^11^. HCCR-1 is upregulated in breast cancer, and HCCR-1 expression is well correlated with prognostic markers, including the presence of ER and PR, p53 mutation and HER2 overexpression^12^. In Ko’s research, transgenic HCCR mice developed metastatic breast cancer. Furthermore, the levels of p21^WAF1^, MDM2, and bax decreased in spontaneous breast cancer and in the metastatic tumours of HCCR transgenic mice^6^. In HCCR-2 transfected lung cancer and colon cancer cells, stabilization of the p53 tumour suppressor occurred without genetic mutation and was correlated with functional impairment^5^. Our previous study indicated that the HCCR-2 gene was abnormally overexpressed in patients with newly diagnosed acute myelogenous leukaemia and acute lymphoblastic leukaemia. In addition, the moment a patient relapsed, the level of HCCR-2 expression increased again^13^. Another study showed that silencing HCCR-2 expression suppressed cell proliferation, induced cell cycle arrest and promoted the apoptosis of K562 cells. Additionally, the expression of bax, p53 and p21 was significantly increased, while Bcl-2 expression was significantly decreased^8^.

STAT3 is also considered to be an oncogene, and its aberrant phosphorylation is most often associated with tumourigenesis. As one of the downstream signalling factors of the interleukin family, STAT3 is considered a point of convergence for numerous oncogenic signalling pathways, and it is constitutively activated in tumour cells^14^,^15^. In particular, STAT3 is known to promote cell proliferation and angiogenesis and to play a role in the invasiveness and metastatic potential of cancers. Other research has shown that STAT3 was activated in human gastric cancer^14^. Previous research demonstrated that STAT3 is a negative regulator of P53 in colorectal cancer. The activation of STAT3 induced the upregulation of MDM2, which is a key negative regulator of P53, and promoted P53 protein degradation^16^. Furthermore, STAT3 is phosphorylated by c-Src and translocates to the nucleus where it binds to the P53 promoter and represses its transcription^17^. In breast cancer, the constitutive activation of STAT3 was shown to attenuate metformin-induced apoptosis^18^. Based on these results, we hypothesized that HCCR overexpression may regulate chemotherapy-induced apoptosis by activating the STAT3 signalling pathway.

In this study we found that gastric cancer cell lines expressed medium or high levels of HCCR compared with expression in the normal gastric epithelial cell line. These data support the hypothesis that HCCR could serve as a diagnostic indicator of gastric cancer. Furthermore, we established a 5-FU resistant HGC-27 subline (HGC-27-R). The western blot and qPCR assays showed that HCCR expression was elevated when cancer cells acquired a resistance to 5-FU. Additional experiments showed that when AGS and HGC-27 cells were treated by relatively low concentrations of 5-FU for a short time, HCCR expression was increased and the cells were resistant to 5-FU-induced apoptosis. These results demonstrate that the enhancement of HCCR expression might be a reason why cancer cells resist chemotherapy and develop 5-FU resistance. Based on the results above, we hypothesized that HCCR might have an anti-apoptosis function. To confirm this hypothesis, we knocked down the HCCR expression of a 5-FU resistant cell subline. After that, the 5-FU-induced apoptotic rate of these cells was increased. This evidence strongly suggests that dysregulated HCCR expression confers 5-FU chemoresistance and that it can rescue cancer cells from 5-FU-induced apoptosis.

We explored the function of HCCR in gastric cancer cell lines by changing their HCCR expression. The MTT assay showed that this expression was positively correlated with the growth rates of the cell lines. FCM analysis indicated that the apoptotic rate was increased when HCCR expression was silenced and vice versa. Additionally, HCCR silencing decreased p-STAT3 expression while increasing the expression of cleaved caspase-3. However, HCCR overexpression induced the opposite results. We did not find any effects of HCCR expression on the expression of P21 and P27 (data not shown). These data suggest that HCCR might not be involved with the progression of the cell cycle but that it may affect cell growth by regulating apoptosis. To test the role that activated STAT3 plays in the progression of apoptosis, we used IL-6 and an STAT3 inhibitor to change the expression of p-STAT3. The results show that apoptosis in cancer cells was decreased when p-STAT3 expression was increased, and vice versa. Thus, we concluded that the apoptosis in cancer cells that is modulated by HCCR expression is dependent on STAT3 activity. Additionally, we found that p-STAT3 expression modulated HCCR expression. In the promoter study, we found two putative STAT3 binding sites in the promoter region of HCCR. When the binding sites of the reporter constructs were mutated, the luciferase activities induced by IL-6 were significantly decreased. Furthermore, the transcriptional activity of the HCCR promoter could be inhibited by the STAT3 inhibitor S3I-201. In summary, the activation of STAT3 is involved in the transcriptional expression of HCCR.

In conclusion, our findings clearly demonstrate that HCCR is an independent biomarker for the diagnosis of gastric cancer and that it regulates apoptosis in cancer cells. Furthermore, HCCR functions as a p-STAT3 stimulator in a positive feedback loop model. In gastric cancer cells, increased HCCR expression confers a more aggressive phenotype and resistance to 5-FU-based chemotherapy.

## Methods and Materials

### Antibodies and reagents

Antibodies against HCCR (Abcam, Cambridge, MA, USA), p27, p21, (p-) STAT3, cleaved caspase-3, (p-) ERK1/2, and (p-) AKT (Cell Signalling Technology, Danvers, MA, USA) were used for western blot and immunohistochemistry. Lipofectamine 2000 Transfection Reagent and the shRNAs were supplied by Invitrogen Life Technologies (San Diego, CA, USA). The reverse transcription-PCR kit was supplied by Promega (Madison, WI, USA). The STAT3 inhibitor S3I-201 was supplied by Santa Cruz Biotechnology (Santa Cruz, CA, USA). All other reagents were of analytical grade or better.

### Cell culture

The human gastric cancer cell lines AGS, MKN-45, BGC823, MGC803, HGC27, SGC7901, NCI-N87 (ATCC) and the normal gastric cell line GES-1 were obtained from the Cancer Institute of the Chinese Academy of Medical Science. All cells were grown in RPMI 1640 medium supplemented with 10% foetal bovine serum and antibiotics. The 5-FU-resistant HGC-27 cell line was selected by continuous exposure to increasing concentrations of 5-FU. 5-FU was added to exponentially growing cultures of HGC-27 cells at a concentration of 0.01 μg/mL and allowed to remain in the culture until cell growth resumed. Over the course of selection, the 5-FU concentration was increased to 2.0 μg/mL. The resulting subline was designated as the HGC-27-R cell line, which was cultured in medium containing 2.0 μg/mL 5-FU.

### Silencing HCCR expression

We used HCG-27 and its subline to perform the HCCR silencing experiment. The 3 different shRNAs (shRNA-1, shRNA-2, shRNA-3) used for the silencing of HCCR have been described in previous articles^8^,^19^. The oligonucleotide sequences used to generate the three different HCCR-targeting shRNAs are listed in Table 1. A scrambled sequence was used as the negative control shRNA. All of these oligonucleotide sequences were sliced and subcloned into *pSuper* vectors. The cells were transfected as described previously. The cell lines were grown in complete RPMI 1640 with G418 (50 mg/mL) during the experiments^20^. The ability of the three transfected HCCR-shRNAs to silence HCCR expression was determined by qPCR and western blot analysis.

### Cell proliferation assay

Cell proliferation was determined by a MTT assay as described previously. Absorbance values at 550 nm were measured with a microplate reader. The results are shown as the mean (±s.d.) absorbance at 550 nm of quadruplicate measurements from six separate experiments^20^.

### Western blot analysis

Total cell lysates were prepared and analysed by western blot as previously described^20^. The specific primary antibodies were used to detect the markers at 4 °C overnight. The blots were visualised using ECL (Promega, Madison, WI, USA) on a Kodak Image Station 4000 mm Pro System (Kodak, Rochester, NY, USA). The density of the bands was quantified by densitometric analysis using the Image Tool (version 3.0) system.

### Quantitative real-time PCR (qPCR)

Total RNA was isolated from tumour cells using Trizol reagent and treated for 45 min at 37 °C with RQ1 DNase (Promega, Madison, WI, USA). The qPCR was performed in an ABI Prism 7500 Sequence Detection System (Applied Biosystems, Beverly, MA, USA). Primers used for the qPCR were as follows: The HCCR primers (AF315598; 1081-1100) were 5′-GGAGGCAGAGAGAGGAGCAG-3′ (sense) and 5′-AGCAAGAGGGTTTGTTTCAGTTCT-3′ (antisense). This primer set amplifies both HCCR-1 and HCCR-2 mRNAs. The primers for GAPDH were 5′-ATGGGGAAGGTGAAGGTCGG-3′ (sense) and 5′-GACGGTGCCATGGAATTTGC-3′ (antisense)^7^.

### Flow cytometry (FCM) analysis of cell apoptosis

Apoptotic cells were detected using an Annexin V-FITC Apoptosis Detection Kit from BioVision (Mountain View, CA, USA). In brief, the cell culture media and the cells were collected and centrifuged. The cells were resuspended in 490 μL of annexin V binding buffer followed by the addition of 5 μL of annexin V-FITC and 5 μL of propidium iodide (PI). The samples were incubated in the dark for 5 min at room temperature and analysed using FCM.

### Reporter plasmid construction and luciferase activity assay

The 1632-bp (from +1419 to +188) upstream region of the HCCR promoter and the 5′-deletion HCCR promoter constructs (including the region from −382 to +188 and the region from −59 to +188) were subcloned into pGL3-basic luciferase plasmids (Promega, Madison, WI, USA). The STAT3 point mutants of −382/+188-Luc were generated using the QuikChange site-directed mutagenesis kit (Stratagene, La Jolla, CA, USA). Cells were seeded prior to cotransfection with the HCCR promoter-pGL3 plasmid constructs, which expressed firefly luciferase, and the pRL-CMV plasmid, which expressed renilla luciferase. Transfected cells were cultured for 24 hours, and the activities of the firefly and renilla luciferases were measured following the protocol of the Dual-Luciferase Reporter Assay System (Promega, Madison, WI, USA) with BioTek Synergy H1 Hybrid Multi-Mode Microplate Readers.

### Xenograft models and immunohistochemistry

The study was approved by the ethics committee of Peking University (Ethics Number: J201155). The experiment was performed in accordance with approved guidelines. Athymic nude mice were randomised to different groups (n = 6 per group). Mice were inoculated subcutaneously in the lower rear flank with human HGC-27 cells transfected by HCCR-shRNA-1 or with negative control shRNA (0.5 × 10^6^ cells per mouse). The tumour volume was calculated (volume = length × width^2^ × 0.52). When the average tumour size reached approximately 50 mm^3^, 5-FU was administered by intraperitoneal injection at a dose of 2 mg/kg, once every other day. At 4 weeks after the start of the 5-FU injections, the primary tumours were excised and paraffin-embedded. Immunostaining analysis was performed to determine the expression of HCCR and p-STAT3. The sections were incubated in HCCR (1:1000 dilution) and p-STAT3 antibodies (1:200 dilution). Images were captured using an Olympus DP71 camera on Olympus BX51 microscopes. To quantify apoptotic cancer cells, sections were incubated with cleaved caspase-3 antibodies (1:100 dilution) followed by Alexa Fluor 555-conjugated secondary antibodies (Invitrogen, San Diego, CA, USA). The number of cleaved caspase-3-positive pixels (red) was divided by the number of DAPI-positive pixels (blue) to approximate the percentage of cells positive for cleaved caspase-3. Immunofluorescent sections were imaged using a Zeiss LSM510 laser scanning confocal microscope. To quantify cancer cells that were positive for HCCR, p-STAT3 and cleaved caspase-3, five random, independent images from 6 mice were scored using IPP (version 6.0, Media Cybernetics, Silver Spring, MD).

### Statistical analysis

The results represent the average of three experiments, and all data are presented as the means ± standard deviation (s.d.). Each experiment was performed at least three times unless otherwise specified. Statistical significance was determined using the unpaired Student’s t-test. P values were two-sided, and a P value less than 0.05 was defined as being statistically significant. Statistical analyses were conducted using the IBM SPSS Statistics software package (version 20, IBM-SPSS Statistics, Armonk, NY, USA).

## Additional Information

**How to cite this article**: Zhang, J.-L. *et al*. Targeting HCCR expression resensitizes gastric cancer cells to chemotherapy via down-regulating the activation of STAT3. *Sci. Rep.* **6**, 24196; doi: 10.1038/srep24196 (2016).

## Figures and Tables

**Table 1:** Sequences used to generate the three synthesized HCCR-targeting shRNAs.

No.	Targeting sequence
shRNA-1	5′-gatccccACAGATCTGTGCACCAAGAttcaagagaTCTTGGTGCACAGATCTGTttttt-3′
	3′-gggTGTCTAGACACGTGGTTCTaagttctctAGAACCACGTGTCTAGACaaaaatcga-5′
shRNA-2	5′-gatccccTAAGATGTGAGAAGCATGGttcaagagaCCATGCTTCTCACATCTTAttttt-3′
	3′-gggATTCTACACTCTTCGTACCaagttctctGGTACGAAGAGTGTAGAATaaaaatcga-5′
shRNA-3	5′-gatccccTTGTGCAGCAAGAGAGACAttcaagagaTGTCTCTCTTGCTGCACAAttttt-3′
	3′-gggAACACGTCGTTCTCTCTGTaagttctctACAGAGAGAACGACGTGTTaaaaatcga-5′

All of these sequences were sliced by the Bgl II and Hind III enzymes and inserted into *pSuper* vectors.

## References

[b1] Ferlay J. . Estimates of worldwide burden of cancer in 2008: GLOBOCAN 2008. Int J Cancer 127, 2893–2917 (2010). 21351269 10.1002/ijc.25516

[b2] Jemal A. . Global cancer statistics. CA Cancer J Clin 61, 69–90 (2011). 21296855 10.3322/caac.20107

[b3] Hartgrink H. H., Jansen E. P., van Grieken N. C. & van de Velde C. J. Gastric cancer. Lancet 374, 477–490 (2009). 19625077 10.1016/S0140-6736(09)60617-6 PMC4613761

[b4] Chung Y. J. & Kim J. W. Novel oncogene HCCR: its diagnostic and therapeutic implications for cancer. Histol Histopathol 20, 999–1003 (2005). 15944950 10.14670/HH-20.999

[b5] Ko J. . Identification and differential expression of novel human cervical cancer oncogene HCCR-2 in human cancers and its involvement in p53 stabilization. Oncogene 22, 4679–4689 (2003). 12879013 10.1038/sj.onc.1206624

[b6] Ko J. . Transgenic mouse model for breast cancer: induction of breast cancer in novel oncogene HCCR-2 transgenic mice. Oncogene 23, 1950–1953 (2004). 14691448 10.1038/sj.onc.1207356

[b7] Jung S. S. . The HCCR oncoprotein as a biomarker for human breast cancer. Clin Cancer Res 11, 7700–7708 (2005). 16278390 10.1158/1078-0432.CCR-04-2609

[b8] Qiao S. K., Ren H. Y., Shi Y. J. & Liu W. Silencing HCCR2 expression inhibits the proliferation of leukemia cells by inducing apoptosis and promoting cell cycle arrest. Int J Mol Med 32, 1373–1379 (2013). 24126909 10.3892/ijmm.2013.1518

[b9] Li M. X. . Prognostic Role of Phospho-STAT3 in Patients with Cancers of the Digestive System: A Systematic Review and Meta-Analysis. PLoS One 10, e0127356 (2015). 26024373 10.1371/journal.pone.0127356 PMC4449159

[b10] Wang Y. C. . CD24 mediates gastric carcinogenesis and promotes gastric cancer progression via STAT3 activation. Apoptosis 19, 643–656 (2014). 24327257 10.1007/s10495-013-0949-9

[b11] Yoon S. K. . The human cervical cancer oncogene protein is a biomarker for human hepatocellular carcinoma. Cancer Res 64, 5434–5441 (2004). 15289352 10.1158/0008-5472.CAN-03-3665

[b12] Ha S. A. . Oncoprotein HCCR-1 expression in breast cancer is well correlated with known breast cancer prognostic factors including the HER2 overexpression, p53 mutation, and ER/PR status. BMC Cancer 9, 51 (2009). 19208263 10.1186/1471-2407-9-51 PMC2672955

[b13] Qiao S. K., Guo X. N., Ren J. H., Zhang J. N. & Wang Y. Quantitative detection of the human cervical cancer oncogene for monitoring the minimal residual disease in acute leukemia. Exp Biol Med 240, 128–134 (2015). 10.1177/1535370214543067 PMC4935186 25034723

[b14] You W. . IL-26 promotes the proliferation and survival of human gastric cancer cells by regulating the balance of STAT1 and STAT3 activation. PLoS One 8, e63588 (2013). 23704922 10.1371/journal.pone.0063588 PMC3660585

[b15] Kohanbash G. & Okada H. MicroRNAs and STAT interplay. Semin Cancer Biol 22, 70–75 (2012). 22210182 10.1016/j.semcancer.2011.12.010 PMC3288787

[b16] Yu H. . LIF negatively regulates tumour-suppressor p53 through Stat3/ID1/MDM2 in colorectal cancers. Nat Commun 5, 5218, doi: 10.1038/ncomms6218 (2014). 25323535 PMC4203416

[b17] Lu Y. . Piwil2 suppresses p53 by inducing phosphorylation of signal transducer and activator of transcription 3 in tumor cells. PLoS One 7, e30999 (2012). 22303479 10.1371/journal.pone.0030999 PMC3267750

[b18] Deng X. S. . Metformin targets Stat3 to inhibit cell growth and induce apoptosis in triple-negative breast cancers. Cell Cycle 11, 367–376 (2012). 22189713 10.4161/cc.11.2.18813

[b19] Guo J. . Silencing of the HCCR2 gene induces apoptosis and suppresses the aggressive phenotype of hepatocellular carcinoma cells in culture. J Gastrointest Surg 15, 1807–1813 (2011). 21796456 10.1007/s11605-011-1633-4

[b20] Zhang J. L. . Secreted protein acidic and rich in cysteine (SPARC) suppresses angiogenesis by down-regulating the expression of VEGF and MMP-7 in gastric cancer. PLoS One 7, e44618 (2012). 22957090 10.1371/journal.pone.0044618 PMC3434168

